# Supplementary material for: Targeting HDAC2-Mediated Immune Regulation to Overcome Therapeutic Resistance in Mutant Colorectal Cancer
Source: Cancers (Basel). 2023 Mar 24;15(7):1960. doi: 10.3390/cancers15071960 (PMC10093005; doi:10.3390/cancers15071960)

Figure S3. Correlation between HDAC2 and related T cell expression in COAD tumors. Scatter plots show the correlation between expression of HDAC2 and (**A**) CD8+ T cells and (**B**) CD4+ T cells. Data shown has been purity-adjusted. Spearman's rho value and statistical significance: positive correlation = $p<0.05$, $\varrho>0$; negative correlation = $p<0.05$, $\varrho<0$; not significant = $p>0.05$.

A

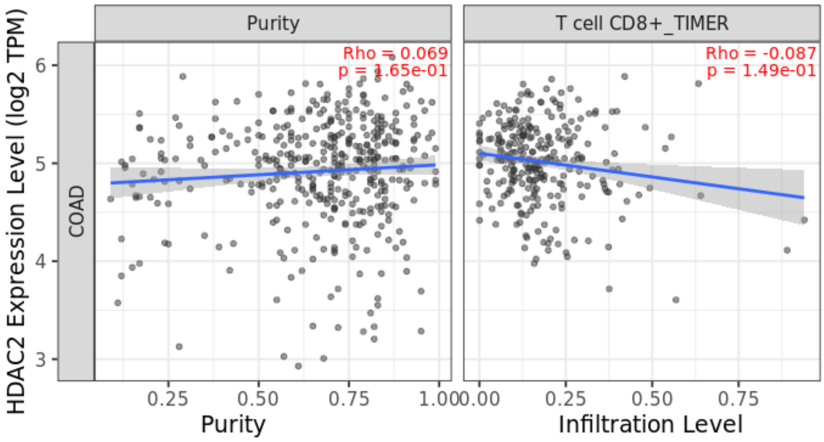

B

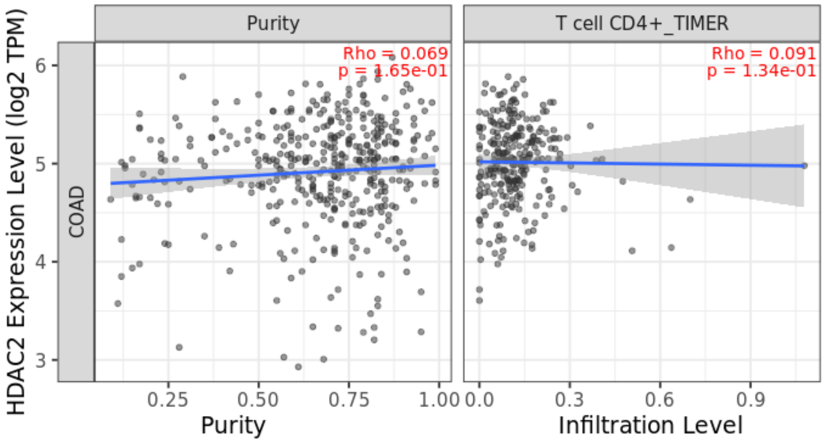

Supplement: Supplementary file 1 [file cancers-15-01960-s001.zip › cancers-2165166-supplementary/Figure S3.pdf]
