# Supplementary material for: Targeting HDAC2-Mediated Immune Regulation to Overcome Therapeutic Resistance in Mutant Colorectal Cancer
Source: Cancers (Basel). 2023 Mar 24;15(7):1960. doi: 10.3390/cancers15071960 (PMC10093005; doi:10.3390/cancers15071960)

Figure S4. Comparison of CNAs in wild-type and altered CRC groups. The left circle (blue) shows CNAs in wild-type samples and the right circle (pink) indicates CNAs in altered samples. Overlapping CNAs are shown in violet.

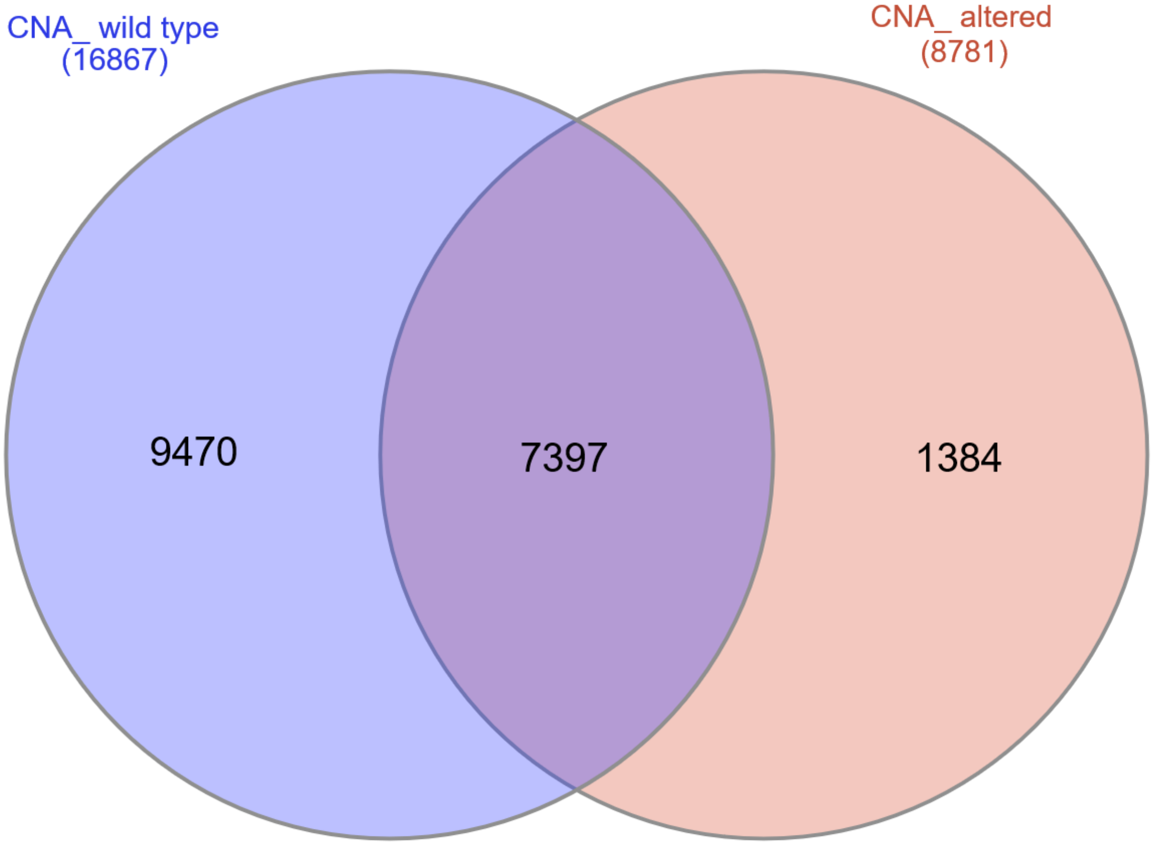

Supplement: Supplementary file 1 [file cancers-15-01960-s001.zip › cancers-2165166-supplementary/Figure S4.pdf]
