# Supplementary material for: Targeting HDAC2-Mediated Immune Regulation to Overcome Therapeutic Resistance in Mutant Colorectal Cancer
Source: Cancers (Basel). 2023 Mar 24;15(7):1960. doi: 10.3390/cancers15071960 (PMC10093005; doi:10.3390/cancers15071960)
Supplement: Supplementary file 1 [file cancers-15-01960-s001.zip › cancers-2165166-supplementary/Table S9 Clinical-pathological features of TMA CRC patients..pdf]

Table S9. Clinical-pathological features of TMA CRC patients

	Clinical-pathological features	Total	Colon dx	Colon sx
n° of patients		44	21	23
age	≤ 65 years	8	4	4
	≥ 65years	36	17	19
sex	M	22	11	10
	F	22	11	12
MSI	Instable	19	11	8
	stable	25	10	15
differentiation grade	1	1	1	0
	2	40	18	22
	3	3	12	1
stadiation	I	14	7	7
	IIA	12	6	6
	IIIA	4	2	2
	IIB	1	0	1
	IIIB	4	3	1
	IIIC	7	3	4
	IV	1	0	1
	N/A	1	0	1
T	0	1	0	1
	1	5	3	2
	2	16	7	9
	3	20	10	10
	4	2	1	1
vascular invasion	absent	34	14	20
	present	3	3	0
	N/A	6	4	2
tumor budding	low grade	5	2	3
	high grade	16	9	7
	N/A	20	9	11
type of proliferation growth	Infiltrative	23	10	13
	Expansive	12	7	5
	N/A	7	3	4

Table S9. Clinical-pathological features of TMA CRC patients

	marked limph type	15	6	9
Inflammation infiltrate	moderate limph type	9	5	4
	mild lymp/monocytic type	2	0	2
	scarce	3	2	1
limph nodes	number	1120	419	701
