# Supplementary material for: Targeting HDAC2-Mediated Immune Regulation to Overcome Therapeutic Resistance in Mutant Colorectal Cancer
Source: Cancers (Basel). 2023 Mar 24;15(7):1960. doi: 10.3390/cancers15071960 (PMC10093005; doi:10.3390/cancers15071960)

Figure S2. Correlation between HDAC2 and CIITA expression in different colon cell lines using the DepMap/CCLE database. Number of points: 73; Pearson: 0.104; Spearman: 0.252; Slope: 9.43E-2; Intercept: -7.58E-3; p-value (linregress): 3.81E-1.

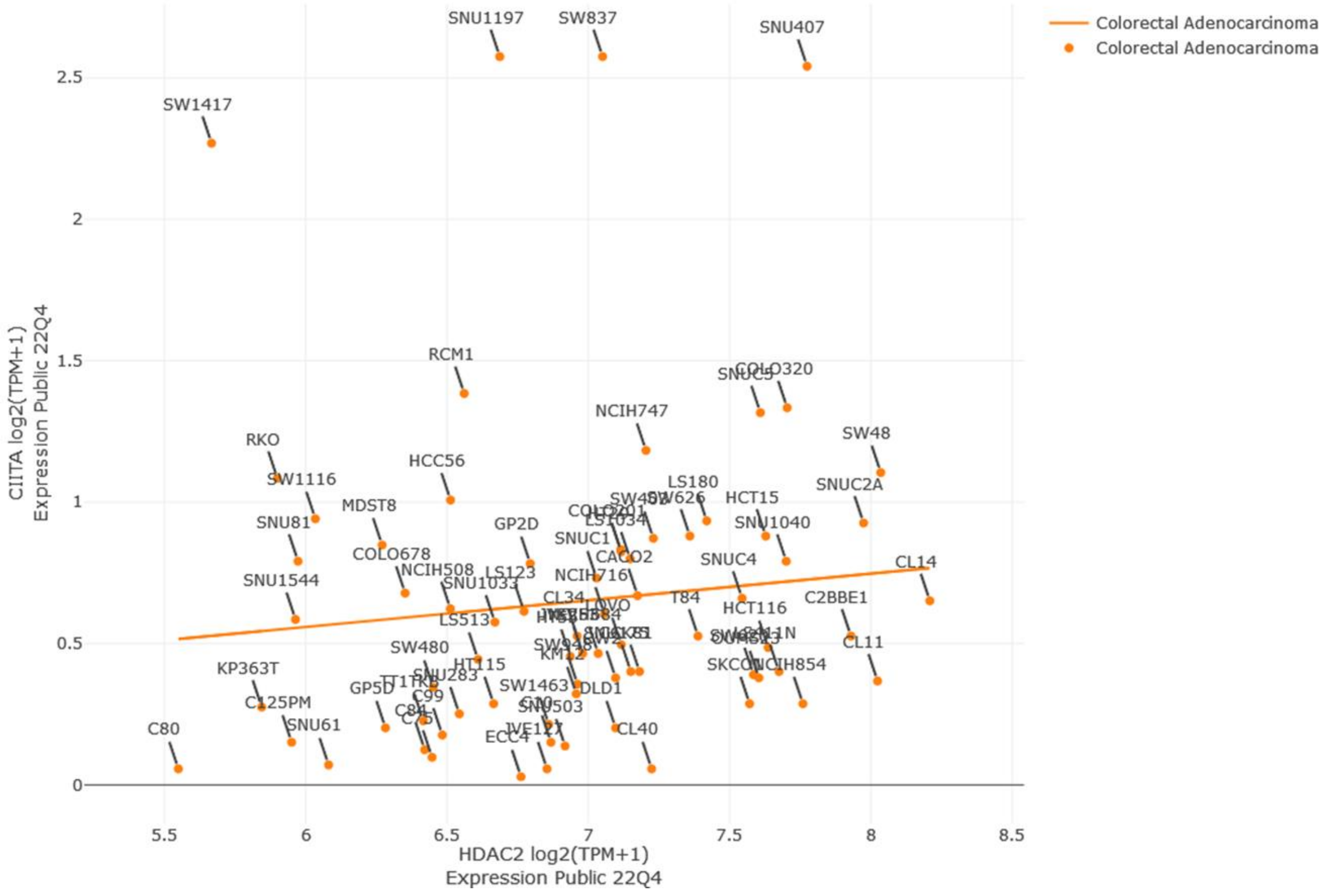

Supplement: Supplementary file 1 [file Figure S2.pdf › cancers-2165166-supplementary/Figure S2.pdf]
